# Reply to van den Broek et al. Comment on “Laratta et al. Marital Stability and Quality of Couple Relationships after Acquired Brain Injury: A Two-Year Follow-Up Clinical Study. *Healthcare* 2021, *9*, 283”

**DOI:** 10.3390/healthcare9081027

**Published:** 2021-08-11

**Authors:** Rocco Salvatore Calabrò, Stefania Laratta, Antonio Cerasa

**Affiliations:** 1 IRCCS Centro Neurolesi Bonino-Pulejo, 98124 Messina, Italy; roccos.calabro@irccsme.it; 2 S. Anna Institute, 88900 Crotone, Italy; s.laratta@isakr.it; 3 Institute for Biomedical Research and Innovation (IRIB), National Research Council of Italy (CNR), 98164 Messina, Italy

We thank van den Broek et al. [1] for their interest in our study and helpful comments. 

As correctly stated by the authors, the main bias of our study is the small sample size that may have affected the statistical correlations between the factors investigated and the couple relationship. However, given the epidemiology of the disease and the lack of larger sample studies on this hot topic, our promising results should be considered as exploratory and deserving confirmation. 

Interesting food for thought by the authors is indeed the potential role of social cognition (SC) in determining the quality of relationships in this patient population [1].

It has been shown that impairment of SC is a neglected but common consequence of acquired brain injury (ABI), and this often leads to disordered interpersonal functioning, including romantic relationships, and poor regulation of personal behavior with impaired social adaptation and quality of life of both the patient and his/her family [2]. In fact, SC is a multidimensional construct that includes a vast number of distinct but interdependent psychological domains, including perceptual functions, such as recognition of emotions, and more elaborate functions, such as empathy, theory of mind (ToM) [3] and pragmatic abilities of communication. These high-order cognitive functions are supported by the activity of several cortical (e.g., prefrontal regions, inferior parietal cortex) and subcortical limbic regions (such as the amygdala) which are impaired in patients with traumatic ABI [3].

Empathy is fundamental to accurately perceive peers’ thoughts, feelings, and other inner mental states. As empathy is often affected in ABI it is important to better evaluate its impact on couple relationships as well as the role of other factors, including educational level and religious practices, as rightly suggested by van den Broek [1]. 

Moreover, as mood disorders and anxiety are also frequent in this patient population [4], and considering that depression may affect SC, a psychiatric evaluation should be also performed to further avoid other biases when assessing couple functioning. 

Finally, sexual functioning is another psychological dimension that should be considered in future studies. Our group has provided evidence [5] about this important factor which provides better management of patient’s functional outcomes and quality of life. In, particular, in a recent narrative review [5], we reported the main findings on the presence of sexual dysfunctions associated with a lower relationship quality, especially in traumatic brain injury patients. Thus, future statistical studies should evaluate the different impacts of sexual dysfunction, SC and sociodemographic characteristics, in order to better define which combination of factors affects couple stability in ABI patients. Obviously, as previously suggested by van den Broek et al. [1], the employment of many demographical and clinical predictors requires the collection of data from larger samples of ABI groups to achieve greater statistical power. Collaboration between different rehabilitative institutes could overcome this problem.
